# Physical Multimorbidity and Social Participation in Adult Aged 65 Years and Older From Six Low- and Middle-Income Countries

**DOI:** 10.1093/geronb/gbab056

**Published:** 2021-03-30

**Authors:** Ruimin Ma, Eugenia Romano, Davy Vancampfort, Joseph Firth, Brendon Stubbs, Ai Koyanagi

**Affiliations:** 1 Department of Psychological Medicine, Institute of Psychiatry, Psychology and Neuroscience (IoPPN), King’s College London, UK; 2 KU Leuven Department of Rehabilitation Sciences, Belgium; 3 University Psychiatric Centre KU Leuven, Kortenberg, Belgium; 4 Division of Psychology and Mental Health, University of Manchester, UK; 5 NICM Health Research Institute, Western Sydney University, Westmead, Australia; 6 Physiotherapy Department, South London and Maudsley NHS Foundation Trust, UK; 7 Research and Development Unit, Parc Sanitari Sant Joan de Déu, Universitat de Barcelona, Fundació Sant Joan de Déu, CIBERSAM, Spain; 8 Institució Catalana de Recerca i Estudis Avancats (ICREA), Barcelona, Spain

**Keywords:** Chronic physical conditions, Comorbidities, Low- and middle-income countries, Multimorbidity, Social participation

## Abstract

**Objectives:**

Multimorbidity is common among older adults from low- and middle-income countries (LMICs). Social participation has a role in protecting against negative health consequences, yet its association with multimorbidity is unclear, particularly in LMICs. Thus, this study investigated the relationship between physical multimorbidity and social participation among older adults across 6 LMICs.

**Method:**

Cross-sectional, community-based data including adults aged 65 years and older from 6 LMICs were analyzed from the WHO Study on Global AGEing and adult health survey. The association between 11 individual chronic conditions or the number of chronic conditions (independent variable) and social participation (range 0–10 with higher scores indicating greater social participation; dependent variable) was assessed by multivariable linear regression analysis.

**Results:**

14,585 individuals (mean age 72.6 [*SD* 11.5] years; 54.9% females) were included. Among individual conditions, hearing problems, visual impairment, and stroke were significantly associated with lower levels of social participation. Overall, an increasing number of chronic conditions was dose-dependently associated with lower levels of social participation (e.g., ≥4 vs 0 conditions: β = −0.26 [95% CI = −0.39, −0.13]). The association was more pronounced among males than females.

**Discussion:**

Older people with multimorbidity had lower levels of social participation in LMICs. Future longitudinal studies are warranted to further investigate temporal associations, and whether addressing social participation can lead to better health outcomes among older people with multimorbidity in LMICs.

Rapid population aging is occurring in low- and middle-income countries (LMICs), and approximately 80% of the older population will be living in LMICs by 2050 (World Health Organization [WHO], 2013). This will inevitably be accompanied by an increase in noncommunicable diseases and multimorbidity in this setting (Afshar et al., 2015). Multimorbidity is defined as the presence of two or more chronic conditions and is an important risk concept due to its association with functional decline (Jindai et al., 2016), poorer quality of life (Peters et al., 2018), increased risk of premature mortality, and health care costs (Kingston et al., 2018). Studies from LMICs have reported a high prevalence of multimorbidity (e.g., 53.8%; Khanam et al., 2011). This is a concern in terms of health care costs, with evidence suggesting that achieving global chronic disease prevention would present an important benefit for the economy of LMICs (Abegunde et al., 2007). Indeed, the prevention of multimorbidity in the older population is becoming a key priority in these regions to avoid further burdening of the economy of LMICs (WHO, 2005).

A potential risk factor for multimorbidity, as well an exacerbating factor of multimorbidity, is social participation. According to a recent content analysis, social participation is mostly defined as a person’s involvement in activities which provide interactions with others in society or the community, and these involvements can happen when taking part in an activity to connect with others or contribute to society, as well as when interacting with others without doing a specific activity with them (Levasseur et al., 2010). Encouraging social participation in the aging population has been highly recommended by the WHO (2002) due to its protective role against chronic conditions (Holmes & Joseph, 2011) such as coronary heart disease (Sundquist et al., 2006) and hypertension (Tu et al., 2018). Previous literature has suggested that the social influence on health could happen through the shaping of social norms, such as encouraging healthier behaviors, as well as through provision of education and information on health (Pellmar et al., 2002), while one review suggested that the beneficial effect of social participation on self-reported health in older adults may be explained by social support and social cohesion within the wider community (Douglas et al., 2017). On the other hand, it is also possible for chronic conditions or multimorbidity to impede social participation, via factors such as limitations in physical function, pain, and discomfort (Bowling, 1995; Zimmer et al., 1997). Thus, it is possible that chronic diseases may lead to lower levels of social participation, and this in turn can lead to further worsening of chronic conditions by depriving patients of information related to health or the social support that they need to treat the chronic conditions.

To date, the few studies on social participation and multimorbidity have yielded mixed results. Some cross-sectional research has acknowledged multimorbidity as a risk factor for lower social participation in older European adults (Galenkamp et al., 2016), and some evidence suggests that symptoms play a key role in predicting social participation restrictions (Griffith et al., 2019). A longitudinal study found a negative association between social participation at baseline and number of chronic conditions developed 4 years later, a relationship mediated by quality of life and depressive symptoms (Santini et al., 2020). However, other studies reported no significant associations on the matter (Alaba & Chola, 2013; Chen et al., 2018; Singer et al., 2019). Given the conflicting results of previous studies and the fact that the majority of these studies have been conducted in high-income countries, clearly more research on this matter is necessary from diverse settings including LMICs.

Studies examining social participation and multimorbidity among older adults are important, as they are one of the most vulnerable populations in terms of access to health information and health care services (Makara, 2013). Furthermore, older individuals are more likely to live in more impoverished areas, lack access to nutritious food, be socially excluded, and experience more daily stress (Makara, 2013), while they require more support for their daily activities as they age (Avlund et al., 2004). These income and social inequalities experienced by older populations may be even more salient in LMICs, where there is presumably limited availability of public infrastructures (e.g., education, social welfare), financial restraints, high unemployment rates, and limited diagnosis and treatment services (Li et al., 2020). Thus, the aim of this study was to examine this association in adults aged 65 years and older from six LMICs (China, Ghana, India, Mexico, Russia, and South Africa), which broadly represent different geographical locations and levels of socioeconomic and demographic transition.

## Method

Data from the Study on Global AGEing and adult health (SAGE) were analyzed. These data are publicly available through http://www.who.int/healthinfo/sage/en/. This survey was undertaken in China, Ghana, India, Mexico, Russia, and South Africa between 2007 and 2010. All countries were LMICs based on the World Bank classification at the time of the survey.

Details of the survey methodology have been published elsewhere (Kowal et al., 2012). In brief, in order to obtain nationally representative samples, a multistage clustered sampling design method was used. The sample consisted of adults aged 18 years and older with oversampling of those aged 50 years and older. Trained interviewers conducted face-to-face interviews using a standard questionnaire. Standard translation procedures were undertaken to ensure comparability between countries. The survey response rates were China: 93%, Ghana: 81%, India: 68%, Mexico: 53%, Russia: 83%, and South Africa: 75%. Sampling weights were constructed to adjust for the population structure as reported by the United Nations Statistical Division. Ethical approval was obtained from the WHO Ethical Review Committee and local ethics research review boards. Written informed consent was obtained from all participants.

### Social Participation Index

As in a previous SAGE publication (Zamora-Macorra et al., 2017), a social participation index was created based on nine questions on the participant’s involvement in community activities in the past 12 months (e.g., attended religious services, club, society, union, etc.) with answer options “never (coded = 1),” “once or twice per year (coded = 2),” “once or twice per month (coded = 3),” “once or twice per week (coded = 4),” and “daily (coded = 5).” The answers to these questions were summed and later converted to a scale ranging from 0 to 10 with higher scores corresponding to higher levels of social participation (Cronbach’s α = 0.79).

### Chronic Conditions and Multimorbidity

We included all 11 chronic physical conditions (angina, arthritis, asthma, chronic back pain, chronic lung disease, diabetes, edentulism, hearing problems, hypertension, stroke, and visual impairment) for which data were available in the SAGE. Chronic back pain was defined as having had back pain every day during the last 30 days. Respondents who answered affirmatively to the question “Have you lost all of your natural teeth?” were considered to have edentulism. The participant was considered to have hearing problems if the interviewer observed this condition during the survey. Hypertension was defined as having at least one of the following: systolic blood pressure ≥140 mmHg, diastolic blood pressure ≥90 mmHg, or self-reported diagnosis. Visual impairment was defined as having severe/extreme difficulty in seeing and recognizing a person that the participant knows across the road (Freeman et al., 2013). Diabetes and stroke were solely based on lifetime self-reported diagnosis. For other conditions, the participant was considered to have the condition in the presence of either one of the following: self-reported diagnosis or symptom-based diagnosis based on algorithms. We used these algorithms, which have been used in previous studies using the same data set, to detect undiagnosed cases (Arokiasamy et al., 2017; Garin et al., 2016). Specifically, the validated Rose questionnaire was used for angina (Rose, 1962), and other previously validated symptom-based algorithms were used for arthritis, asthma, and chronic lung disease (Arokiasamy et al., 2017). Further details on the definition of chronic physical conditions can be found in Supplementary Table S1. The total number of chronic physical conditions was calculated and categorized as no chronic conditions or one, two, three, and four or more chronic conditions. Multimorbidity was defined as having two or more chronic physical conditions, in line with previously used definitions (Garin et al., 2016).

### Control Variables

The control variables were selected based on past literature (Kristensen et al., 2019), and included age, sex, wealth quintiles based on income, level of highest education achieved, marital status (married/cohabiting, never married, separated/divorced/widowed), living arrangement (alone or not), body mass index (BMI), physical activity, smoking (never, current, former), alcohol consumption (never, nonheavy, heavy), loneliness, and depression. BMI (kg/m^2^) was based on measured weight and height and was categorized as: <18.5 (underweight), 18.5–24.9 (normal weight), 25.0–29.9 (overweight), and ≥30.0 (obese). Physical activity levels were assessed with the Global Physical Activity Questionnaire (Bull et al., 2009). The total amount of moderate-to-vigorous physical activity in a typical week was calculated based on self-report. Those scoring ≥150 min of moderate-to-vigorous intensity physical activity were classified as meeting the recommended guidelines (coded = 0), and those scoring <150 min (low physical activity) were classified as not meeting the recommended guidelines (coded = 1) (WHO, 2010). Consumers of at least four (females) or five drinks (males) of any alcoholic beverage per day on at least 1 day in the past week were considered to be “heavy” drinkers. Those who had ever consumed alcohol but were not heavy drinkers were categorized as “nonheavy” drinkers (Koyanagi et al., 2015). Loneliness was assessed with the question “Did you feel lonely for much of the day yesterday?” with answer options “yes” or “no.” Questions based on the World Mental Health Survey version of the Composite International Diagnostic Interview (Kessler & Üstün, 2004) were used for the endorsement of DSM-IV depression (American Psychiatric Association, 2000).

### Statistical Analysis

The statistical analysis was performed with Stata 14.1 (Stata Corp LP, College station, TX). The analysis was restricted to those aged 65 years and older. The difference in sample characteristics between those with and without multimorbidity (i.e., two or more chronic physical conditions) was tested by chi-squared tests and Student’s *t* tests for categorical and continuous variables, respectively. Multivariable linear regression analysis was conducted to assess the association between the individual 11 chronic physical conditions or number of chronic physical conditions (independent variable) and the social participation index score (dependent variable). In order to assess whether the association between the number of chronic physical conditions and social participation differs by sex, we tested for interaction by sex by including an interaction term (Number of chronic physical conditions × Sex) in the model. Because preliminary analysis showed that there is a significant interaction by sex, we stratified the analysis by sex for this analysis.

All regression analyses were adjusted for age, sex, wealth, education, marital status, living arrangement, BMI, physical activity, smoking, alcohol consumption, loneliness, depression, and country, except for the sex-stratified analysis which was not adjusted for sex. For the analysis on individual chronic conditions, all conditions were included simultaneously in the model. Adjustment for country was done by including dummy variables for each country in the model as in previous SAGE publications (Koyanagi et al., 2019). All variables were included in the models as categorical variables with the exception of age and the social participation index score (continuous variables). The sample weighting and the complex study design were taken into account in all analyses. Results from the regression analyses are presented as *b* coefficients with 95% confidence intervals. The level of statistical significance was set at *p* < 0.05.

## Results

**Figure 1.**
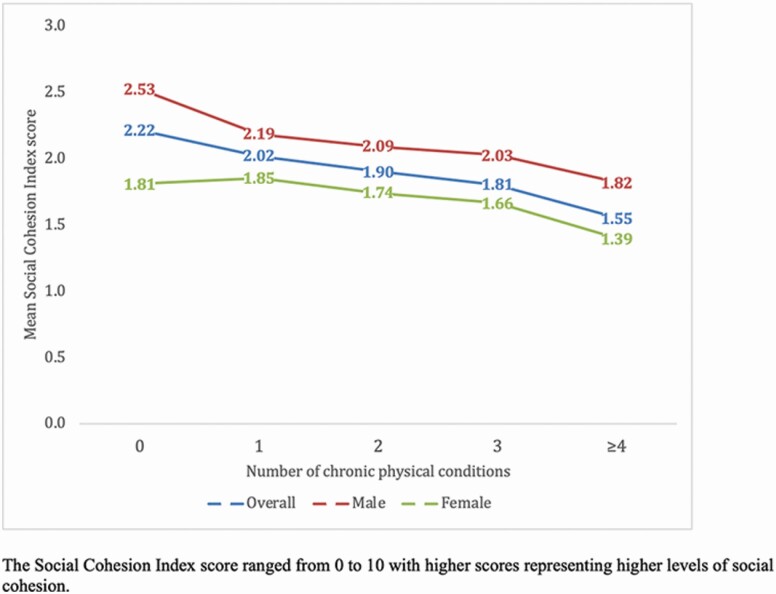
Mean social participation index score by number of chronic physical conditions (overall and by sex).

**Figure 2.**
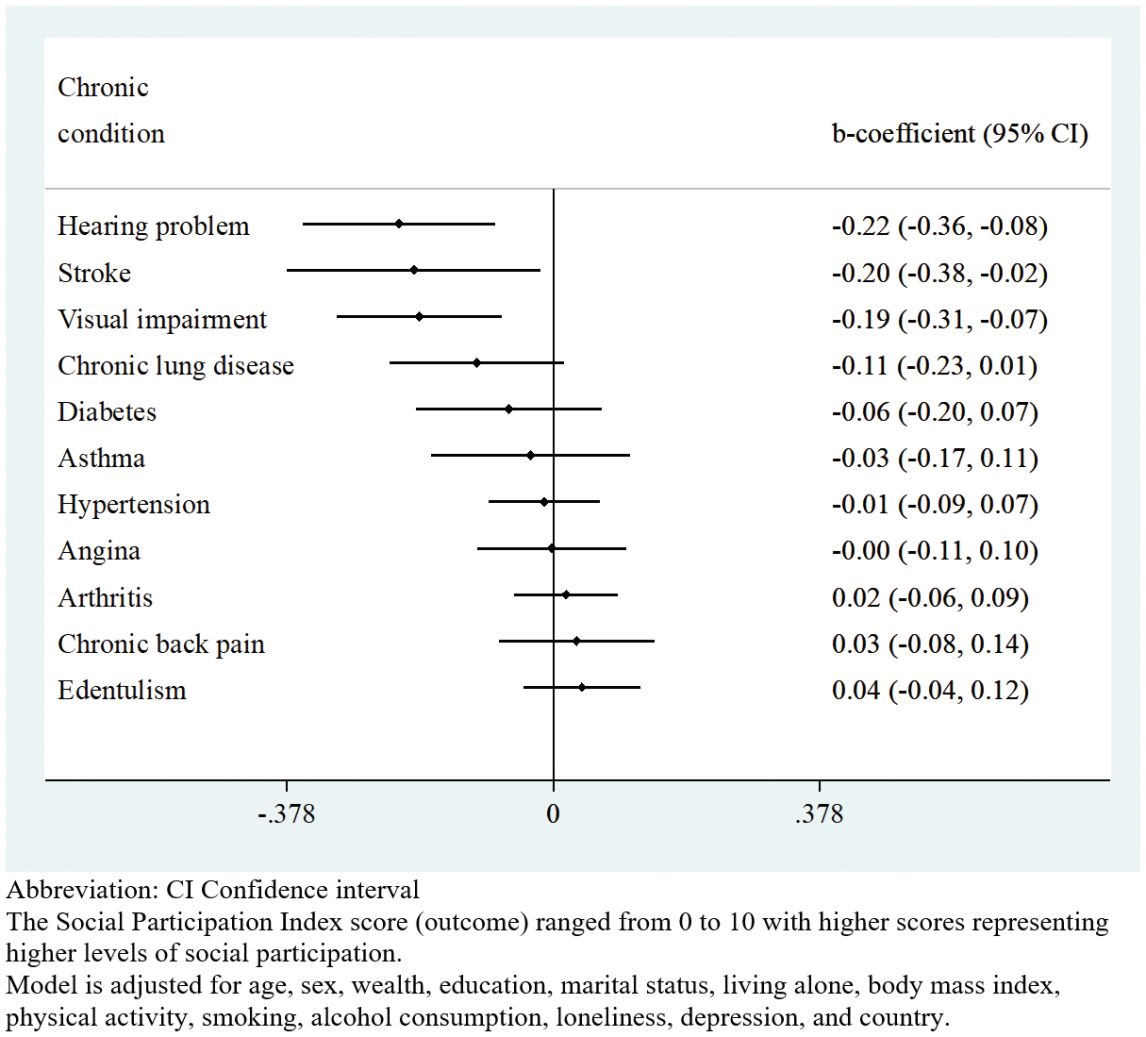
Association between individual chronic physical conditions and social participation index score estimated by multivariable linear regression.

The final sample included 14,585 individuals aged 65 years and older (5,360: China; 1,975: Ghana; 2,441: India; 1,375: Mexico; 1,950: Russia; 1,484: South Africa). The sample characteristics are provided in Table 1. The mean (*SD*) age was 72.6 (11.5) years, while 54.9% were females. The frequency of each social activity included in the social participation index by each country is reported in Supplementary Table S2. The prevalence of different types of chronic conditions by sex is shown in Table 2. The level of social participation, as expressed in terms of the mean social participation index score, was lower among those with a greater number of chronic physical conditions (Figure 1). The association between the individual chronic physical conditions and the social participation index score estimated by multivariable linear regression is shown in Figure 2. Significantly lower levels of social participation were observed for those with hearing problems, visual impairment, and stroke. In terms of the number of chronic physical conditions, overall, levels of social participation decreased with increasing number of chronic physical conditions (Table 3). However, when the analyses were stratified by sex, only four or more (vs no chronic conditions) chronic physical conditions were significantly associated with lower levels of social participation among women, while for men, one to four chronic conditions were all significantly associated with lower levels of social participation.

**Table 1.** Sample Characteristics (Overall and by Physical Multimorbidity)

	Physical multimorbidity^a^			
Characteristic	Overall	No	Yes	*p* Value^b^
Social participation index score^c^, mean (*SD*)	1.85 (2.39)	2.08 (2.49)	1.76 (2.27)	<.001
Age (years), mean (*SD*)	72.6 (11.5)	71.2 (10.6)	73.2 (11.5)	<.001
Sex				
Male	45.1	52.8	40.6	<.001
Female	54.9	47.2	59.4	
Wealth				
Poorest	21.8	20.3	22.6	.019
Poorer	21.0	19.5	21.9	
Middle	20.4	19.1	21.1	
Richer	17.4	18.3	16.8	
Richest	19.5	22.8	17.6	
Education				
Primary	63.5	65.4	62.4	.172
Secondary	30.0	27.4	31.5	
Tertiary	6.5	7.1	6.1	
Marital status				
Married/cohabiting	60.8	67.8	56.8	<.001
Never married	1.2	1.1	1.3	
Separated/divorced/widowed	38.0	31.2	41.9	
Living alone				
No	83.5	87.7	81.1	<.001
Yes	16.5	12.3	18.9	
Body mass index (kg/m^2^)				
18.5–24.9	46.3	52.5	42.7	<.001
25.0–29.9	23.6	20.2	25.6	
≥30	10.3	7.2	12.2	
<18.5	19.7	20.2	19.5	
Low physical activity				
No	65.7	71.5	62.4	<.001
Yes	34.3	28.5	37.6	
Smoking				
Never	62.0	59.7	63.3	.001
Current	29.5	33.2	27.4	
Former	8.5	7.1	9.3	
Alcohol consumption				
Never	67.7	70.0	66.3	.095
Nonheavy	30.0	27.3	31.6	
Heavy	2.4	2.7	2.1	
Loneliness				
No	85.5	92.1	81.7	<.001
Yes	14.5	7.9	18.3	
Depression				
No	93.4	98.3	90.6	<.001
Yes	6.6	1.7	9.4	

*Notes*: Data are % unless otherwise stated.

^a^Physical multimorbidity referred to two or more chronic physical conditions. ^b^*p* Value was calculated by Student’s *t* tests and chi-squared test for continuous and categorical variables, respectively. ^c^The social participation index score ranged from 0 to 10 with higher scores representing higher levels of social participation.

**Table 2.** Prevalence of Individual Chronic Conditions (Overall and by Sex)

Chronic condition	Overall	Male	Female
Angina	24.2	19.5	28.1
Arthritis	35.0	28.7	40.3
Asthma	10.0	11.7	8.6
Chronic back pain	11.2	7.7	14.1
Chronic lung disease	21.1	21.8	20.5
Diabetes	8.6	8.0	9.1
Edentulism	22.6	19.8	25.0
Hearing problem	10.7	10.9	10.6
Hypertension	63.4	58.2	67.6
Stroke	4.6	5.3	4.1
Visual impairment	12.9	9.8	15.4

*Note*: Data are %.

**Table 3.** Association of Number of Chronic Physical Conditions and Covariates With Social Participation Index Score Estimated by Multivariable Linear Regression

	Overall		Male		Female	
Characteristic	*b*	95% CI	*b*	95% CI	*b*	95% CI
Number of chronic physical conditions						
0	Ref.		Ref.		Ref.	
1	−0.12*	[−0.24, −0.00]	−0.26**	[−0.43, −0.09]	0.07	[−0.09, 0.23]
2	−0.13*	[−0.25, −0.02]	−0.22*	[−0.39, −0.04]	0.04	[−0.09, 0.17]
3	−0.15*	[−0.29, −0.00]	−0.20*	[−0.38, −0.02]	0.01	[−0.19, 0.20]
4	−0.26***	[−0.39, −0.13]	−0.27**	[−0.46, −0.08]	−0.15*	[−0.29, −0.01]
Age (years)						
Per 1-year increase	−0.02***	[−0.03, −0.01]	−0.02***	[−0.03, −0.02]	−0.02***	[−0.03, −0.01]
Sex						
Male	Ref.					
Female	−0.31***	[−0.44, −0.18]				
Wealth						
Poorest	Ref.		Ref.		Ref.	
Poorer	0.22**	[0.07, 0.38]	0.17	[−0.00, 0.35]	0.26**	[0.08, 0.45]
Middle	0.19**	[0.05, 0.32]	0.18*	[0.01, 0.36]	0.19*	[0.03, 0.34]
Richer	0.29***	[0.16, 0.43]	0.36***	[0.20, 0.53]	0.24**	[0.07, 0.41]
Richest	0.36***	[0.22, 0.50]	0.49***	[0.30, 0.69]	0.23**	[0.08, 0.38]
Education						
Primary	Ref.		Ref.		Ref.	
Secondary	0.14*	[0.03, 0.26]	0	[−0.14, 0.14]	0.17*	[0.03, 0.32]
Tertiary	0.28**	[0.08, 0.49]	0.16	[−0.10, 0.41]	0.32*	[0.06, 0.57]
Marital status						
Married/cohabiting	Ref.		Ref.		Ref.	
Never married	−0.36**	[−0.59, −0.14]	−0.73***	[−1.14, −0.33]	−0.18	[−0.43, 0.06]
Separated/divorced/widowed	−0.19**	[−0.31, −0.06]	−0.15*	[−0.31, −0.00]	−0.06	[−0.20, 0.07]
Living alone						
No	Ref.		Ref.		Ref.	
Yes	0.35***	[0.23, 0.47]	0.29**	[0.08, 0.51]	0.21**	[0.08, 0.34]
Body mass index (kg/m^2^)						
18.5–24.9	Ref.		Ref.		Ref.	
25.0–29.9	−0.03	[−0.12, 0.05]	−0.04	[−0.16, 0.08]	−0.02	[−0.12, 0.09]
≥30	−0.03	[−0.19, 0.12]	0.09	[−0.15, 0.34]	−0.12	[−0.29, 0.05]
<18.5	−0.03	[−0.15, 0.09]	−0.02	[−0.18, 0.14]	−0.04	[−0.19, 0.12]
Low physical activity						
No	Ref.		Ref.		Ref.	
Yes	−0.38***	[−0.48, −0.29]	−0.45***	[−0.58, −0.33]	−0.33***	[−0.42, −0.23]
Smoking						
Never	Ref.		Ref.		Ref.	
Current	−0.13*	[−0.22, −0.03]	−0.17*	[−0.30, −0.04]	−0.04	[−0.18, 0.10]
Former	−0.04	[−0.18, 0.09]	0.01	[−0.15, 0.18]	0.11	[−0.17, 0.39]
Alcohol consumption						
Never	Ref.		Ref.		Ref.	
Nonheavy	0.05	[−0.09, 0.20]	0.04	[−0.07, 0.16]	0.07	[−0.16, 0.30]
Heavy	0.30**	[0.08, 0.52]	0.47***	[0.24, 0.71]	0.15	[−0.20, 0.50]
Loneliness						
No	Ref.		Ref.		Ref.	
Yes	−0.19***	[−0.29, −0.09]	−0.19*	[−0.37, −0.02]	−0.22***	[−0.33, −0.10]
Depression						
No	Ref.		Ref.		Ref.	
Yes	−0.01	[−0.18, 0.16]	−0.13	[−0.36, 0.11]	0.08	[−0.14, 0.31]

*Notes*: CI = confidence interval; Ref. = reference category. The social participation index score (outcome) ranged from 0 to 10 with higher scores representing higher levels of social participation. Models are adjusted for all variables in the respective columns and country.

*p < .05. **p < .01. ***p < .001.

## Discussion

### Main Findings

To the best of our knowledge, this is the first study to investigate the association between social participation and multimorbidity among older adults from LMICs. Our results demonstrate a concerning linear decrease in the level of social participation with increasing number of chronic conditions. Significant sex differences were also observed, with having any number of chronic conditions being significantly associated with lower levels of social participation among men, whereas among women, only four or more chronic conditions were significantly associated with lower levels of social participation. These study results highlight the importance of taking social participation into consideration in public health interventions to tackle multimorbidity, and its health and social consequences in LMICs.

### Interpretation of Findings

In terms of individual chronic conditions, hearing problems, visual impairment, and stroke were significantly associated with lower levels of social participation in our study. The lower level of social participation found in people with hearing problems and visual impairment may be explained by the limited ability of people with these conditions to establish social ties as vision and hearing abilities are essential for communication. For example, visual impairment in later life has been associated with increased social isolation and diminished social skills (Thurston et al., 2010), while hearing loss or impairment negatively impact the quality of one’s social life, especially for older adults, due to associated psychosocial consequences of hearing loss and diminished ability in understanding speech (Picinini et al., 2017). One survey of seniors aged 65 and over reported a significantly lower level of social participation in seniors with visual impairment, compared to those without, even after adjusting a range of covariates (Jin et al., 2019). In terms of stroke, the associated physical disabilities and psychosocial problems (e.g., fatigue, depression, and anxiety) may hinder social interaction poststroke (Bergersen et al., 2010; Hackett et al., 2014; Pan et al., 2011), as the limited mobility accounted by this condition may restrict social participation especially outside of home.

In the current study, social participation was associated with several individual chronic conditions and multimorbidity. This is in line with previous research, which has found that older adults with health difficulties tend to report poor engagement in social activities (Strain et al., 2002). In particular, diminished functional ability (Bowling, 1995), severity of physical conditions, and associated pain and discomfort could contribute to poor social participation (Zimmer et al., 1997). However, we may speculate that the association between social participation and multimorbidity is bidirectional. In a meta-analysis determining the relationship between social relationships and the risk for mortality, the authors found a 50% increased likelihood of survival for those with stronger social relationships even after adjustment for a variety of confounders, and the impact of social relationships on mortality was found to be comparable with a number of lifestyle risk factors, including smoking, alcohol consumption, and BMI (Holt-Lunstad et al., 2010). Based on the buffer effect model (Cohen, Gottilieb, and Underwood, 2000), social participation could potentially buffer against the negative impact from life stressors, and thus impede the detrimental effect of social stressors on physical health. It is also possible that regular and active social participation could motivate a healthy lifestyle, including more physical activities, maintaining a healthy weight, and seeking health care when in need. Given this, regardless of temporal associations, the mere fact that individuals with multimorbidity have lower levels of social participation may be an issue since they may be exposed to more stressors, lack information on how to maintain a healthy lifestyle, or lack support that is necessary to treat their chronic diseases, and this may lead to worsening in health status.

In our study, social participation was found to be negatively associated with loneliness, but surprisingly positively linked to living alone. One explanation could be that for those who live alone, they may attempt to maintain their level of social engagement and social relationships with others by increasingly participating in social activities or events that are outside their households. Most importantly, our results also underline that, although low social participation could cause loneliness (Pettigrew & Roberts, 2008), the relationship between living alone and social participation is not the same as that of loneliness, hence living alone should not be considered synonymous as loneliness. Loneliness is an unpleasant experience that occurs when there is a mismatch between a person’s desired and perceived availability and quality of social interactions/relationships (Peplau & Perlman, 1982), and a systematic review of loneliness interventions (Ma et al., 2020) has emphasized that simply increasing social opportunities or social participation is not an effective approach in improving loneliness. Therefore, our study further highlights the importance that living alone and loneliness should not be used interchangeably in literature, and strategies to address loneliness specifically should be warranted.

The association between multimorbidity and lower social participation was also stronger in men than women. This sex difference was unlikely to be explained by the different patterns of chronic conditions among men and women as conditions that are particularly strongly associated with lower levels of social participation (e.g., hearing problem, visual impairment, stroke) were not more prevalent among men. Although the reasons for this finding can only be speculated, several mechanisms may be suggested. First, compared to men, women in LMICs may be more confined in their traditional gender role, for example, taking care of (grand-)children and domestic chores (Kuper & Marmot, 2003) and be more likely to have had social interaction at home throughout their life. In this case, developing diseases that hinder leaving the house may have less impact on their level of social participation. Men in LMICs, on the other hand, may be more likely to work full-time, even at an older age, and chronic health conditions may therefore limit their opportunities to engage in regular work activities or interact with people at work, further affecting their level of social participation. An alternative explanation for this pattern could be that women are less likely to lose their social contacts even when their mobility is impacted by chronic physical conditions, as they tend to have a larger social network and more social contacts with their children and friends compared to men (Beach & Bamford, 2014). This greater social connectedness of women may be reflected in the findings of our study where we found that social participation was negatively linked to being separated/divorced/widowed and never married in men, but such relationship was not significant in women. Finally, men and women may cope with life stressors differently. When facing decreased social participation in their social environment or stressors from workplace, men may be more likely to engage in prolonged high effort coping in order to overcome these perceived barriers in their lives (Subramanyam et al., 2013), and this type of coping strategy may lead to negative health outcomes (James et al., 1992).

Another interesting sex difference that we found was that higher education levels were significantly associated with greater levels of social participation only among women but not men. This may be because women with low levels of education are more likely to engage in their traditional caring role at home, while this may not be the case in men. This suggests that education among women may protect them from being socially isolated, as this may enable them to increase their chances of obtaining a job and consequently have a more diverse social network consisting of friends, family, clients, and colleagues at work.

### Policy Implications

The current study reports several significant findings on multimorbidity and social participation, with important implications for future research and clinical practice. In particular, these results have crucial implication in LMICs, where expenditure for health care may be highly burdensome especially in countries without universal health insurance schemes, even leading to catastrophic health expenditure (Kinfu et al., 2009). Facilitating social participation should be recognized as an ultimate goal at a national level, in order to buffer against increased income disparities and health inequalities in LMICs (Hu et al., 2014). Multidimensional initiatives, including those focusing on social (e.g., cultural recreation, volunteering opportunities), psychosocial (e.g., well-being, quality of life), and material (e.g., access to public transportation) could also be broadly introduced, all of which could be significant contributors to successful ageing (WHO, 2002).

Another key implication is that health care providers should be mindful about those populations with a high likelihood of poor social participation, for example, those who suffer from stroke, visual impairment, hearing problems, and most importantly, people with multimorbidity. By collaborating with the government, health care providers should recognize any difficulties that may hinder social participation (e.g., reduced physical function, financial difficulties, housing problems, poor transportation) in people with multimorbidity and certain physical conditions and refer them to relevant social services or introduce them to community-based programs during routine care (e.g., peer support groups and befriending programs), with the potential to improve social participation among patient groups.

### Strengths and Limitations

This is the first study addressing the existing knowledge gap in terms of the association between social participation and multimorbidity among older people from LMICs. This is in line with the WHO Commission on Social Determinants of Health framework (2009), which emphasizes the inadequacy of current focus on biological or physical factors as singular determinants of health. The strength of the study includes the large sample size and the use of nationally representative data sets. However, several limitations should be considered. First, the evaluation of chronic conditions was mostly based on self-reported measures, which may potentially lead to reporting bias. Second, although this study included a list of chronic conditions common in old age, we lacked data on diseases such as cancer. Thus, the results may differ with more chronic diseases being included. Third, there is no conventional way of assessing social participation, but it is a common method to construct participation variables from summary participation indices (Cohen, Underwood, et al., 2000). Lastly, the cross-sectional nature of the current study hampered our interpretation of causality and temporality between social participation and physical multimorbidity. Therefore, future longitudinal studies are warranted to further investigate temporal associations, as well as the mechanisms by which social participation may impact physical multimorbidity or vice versa.

## Conclusions

In summary, the results of our study on older adults from six LMICs suggest that low social participation is associated with multimorbidity. Although the temporal association could not be established in our study, the mere fact that people with multimorbidity are more likely to have lower levels of social participation is problematic, as both multimorbidity and low social participation are associated with adverse outcomes, while it is possible that low levels of social participation may exacerbate multimorbidity. Enhancing social participation in people with multimorbidity may create a sense of belonging and resilience (Choi & Matz-Costa, 2018), enhance access to leisure activities and health care services (Fone et al., 2007), strengthen emotional and instrumental support, make older people feel that they are loved and being cared for (Jen et al., 2010), and ultimately promote successful and healthy ageing (Choi & Matz-Costa, 2018). Future studies should investigate how social participation can be promoted among people with multimorbidity, while studies on whether the promotion of social participation may lead to a reduction in multimorbidity are also warranted.

## Supplementary Material

gbab056_suppl_Supplemental_Material
